# Seasonality of respiratory viruses and bacterial pathogens

**DOI:** 10.1186/s13756-019-0574-7

**Published:** 2019-07-22

**Authors:** Young June Choe, Michael A. Smit, Leonard A. Mermel

**Affiliations:** 1 Department of Pediatrics, Warren Alpert Medical School of Brown University, Providence, Rhode Island USA; 2 Division of Infectious Diseases, Children’s Hospital Los Angeles and Keck School of Medicine, University of Southern California, Los Angeles, California USA; 3 Department of Epidemiology and Infection Control, Rhode Island Hospital, and Department of Medicine, Warren Alpert Medical School of Brown University, Providence, Rhode Island USA; 4 Division of Infectious Diseases, Rhode Island Hospital, 593 Eddy Street, Providence, RI 02903 USA

**Keywords:** Seasonality, Trend, Respiratory virus, Bacteria, *C. difficile*, MRSA

## Abstract

**Background:**

Seasonal variation has been observed for various bacterial and viral infections. We aimed to further study seasonality of respiratory viruses and bacterial pathogens in relation to antibiotic use, as well as meteorological parameters.

**Methods:**

An ecologic study of antibiotic exposure, meteorological parameters, detection of respiratory viruses  and clinical isolates of *Clostridioides difficile*, Methicillin-resistant *Staphylococcus aureus* (MRSA), *Streptococcus pneumoniae*, and *Escherichia coli* and *Klebsiella pneumoniae* (grouped together as gram-negative bacteria; GNB) in Rhode Island from 2012 to 2016.

**Results:**

Peak detection of *C. difficile* occurred 3 months after the peak in antibiotic prescriptions filled (OR = 1.24, 95% CI, 1.07–1.43; *P* = 0.006). Peak MRSA detection was noted 7 months after the peak in antibiotic prescriptions filled (OR = 1.69, 95% CI, 1.21–2.35; *P* = 0.003) and 10 months after the peak in respiratory virus detection (OR = 1.04, 95% CI, 1.01–1.06; *P* = 0.003). Peak GNB detection was noted 2 months after the peak mean monthly ambient temperature (OR = 1.69, 95% C.I., 1.20–2.39; *P* = 0.004). Peak detection of *S. pneumoniae* was noted at the same time as the peak in detection of respiratory viruses (OR = 1.01, 95% C.I., 1.00–1.01; *P* = 0.015).

**Conclusions:**

We identified distinct seasonal variation in detection of respiratory viruses and bacterial pathogens. *C. difficile* seasonality may, in part, be related to antibiotic prescriptions filled; GNB seasonality may be related to ambient temperature and *S. pneumoniae* may be related to concurrent respiratory viral infections.

**Electronic supplementary material:**

The online version of this article (10.1186/s13756-019-0574-7) contains supplementary material, which is available to authorized users.

## Background

Many host-related risk factors have been established for bacterial infections; however, non-intrinsic factors such as respiratory viral infections, weather conditions, and latitude are among other factors that lead to seasonality of such infections [1–6]. In addition, *Clostridioides difficile* infection is temporally associated with respiratory tract infections [6–8], likely due to antibiotic overprescribing for respiratory viral infections.

A better understanding of seasonality of various pathogens, as well as an association with antibiotic prescriptions may allow us to devise and implement future public health interventions (e.g., vaccination and antimicrobial stewardship), particularly in the community setting. The purpose of this study was to assess seasonality of respiratory viruses and bacterial pathogens in relation to antibiotic use and meteorological parameters in one locale.

## Methods

### Study setting

This was an ecological study involving detection of bacterial pathogens and respiratory viruses in Rhode Island. Microbiologic data included specimens sent to the Lifespan Microbiology Laboratory from inpatients and outpatients assessed in the  Lifespan healthcare system (including Rhode Island Hospital/Hasbro Children’s Hospital, Miriam Hospital, Newport Hospital, Bradley Hospital, and Gateway Healthcare). The system is licensed for 1,165 beds, with 62,100 patient discharges, 257,081 emergency department visits, and 757,380 outpatient visits [9], as well as patient specimens from other outpatient facilities throughout Rhode Island between January 1, 2012 and December 31, 2016. We also obtained data regarding antibiotics prescriptions, total precipitation and average temperature.

### Data source for detection of respiratory viruses and bacterial pathogens

Rhode Island Hospital infection control software system (TheraDoc; Premier, Charlotte, NC) was queried to find positive microbiology laboratory identification of respiratory viruses identified by the respiratory viral panel (Luminex, Austin, TX), rapid influenza test (Xpert; Cepheid, Sunnyvale, CA) or rapid Respiratory Syncytial Virus test (Xpert; Cepheid, Sunnyvale, CA) from an outpatient’s or inpatient’s nasopharyngeal swab or bronchoscopic lavage specimen. The target population included all ages regardless of insurance status. The respiratory viral panel included testing for Influenza A/B, respiratory syncytial virus (RSV) A/B, Coronavirus, Parainfluenza virus, human Metapneumovirus, Adenovirus, Rhinovirus/Enterovirus (the panel does not differentiate Rhinovirus and Enterovirus). *Clostridioides difficile* toxin was identified by PCR testing of stool specimens (BD GeneOhm; Xpert); growth in clinical cultures identified cases involving Methicillin-resistant *Staphylococcus aureus* (MRSA), *Streptococcus pneumoniae*, and *Escherichia coli* and *Klebsiella pneumoniae* (grouped together as gram-negative bacteria; GNB). Only the first encounter with one of the above-noted pathogens was included if a patient had multiple episodes of positive laboratory testing for that pathogen within 30 days. We excluded MRSA identified only from nares screening. Incidence reflected counts with no denominator based on the assumption that there was no significant year-to-year variability between practices in obtaining respiratory specimens (Additional file 1: Figure S1).

### Antibiotics and meteorological data source

Antibiotic prescription data from January 2012 through December 2016 was used to assess antibiotic exposure. Data was obtained from the Rhode Island All Payer Claim Database (ACPD), provided by Rhode Island Department of Health. The ACPD collects and stores payer enrollment data, medical claims, pharmacy claims, and provider data on a monthly basis from commercial insurers, Medicare and Medicaid regarding antibiotic prescriptions filled [10]. The denominator for prescriptions per 1,000 population was derived from Rhode Island census data [11]. Antibiotic classes were: cephalosporins, clindamycin, macrolides, metronidazole, nitrofurantoin, penicillins, quinolones, tetracyclines, and trimethoprim-sulfamethoxazole.

Monthly aggregated data on average temperature and total precipitation from January 2012 through December 2016 in Rhode Island was retrieved from the National Centers for Environmental Information [12].

### Data analysis

**Fig. 1:**
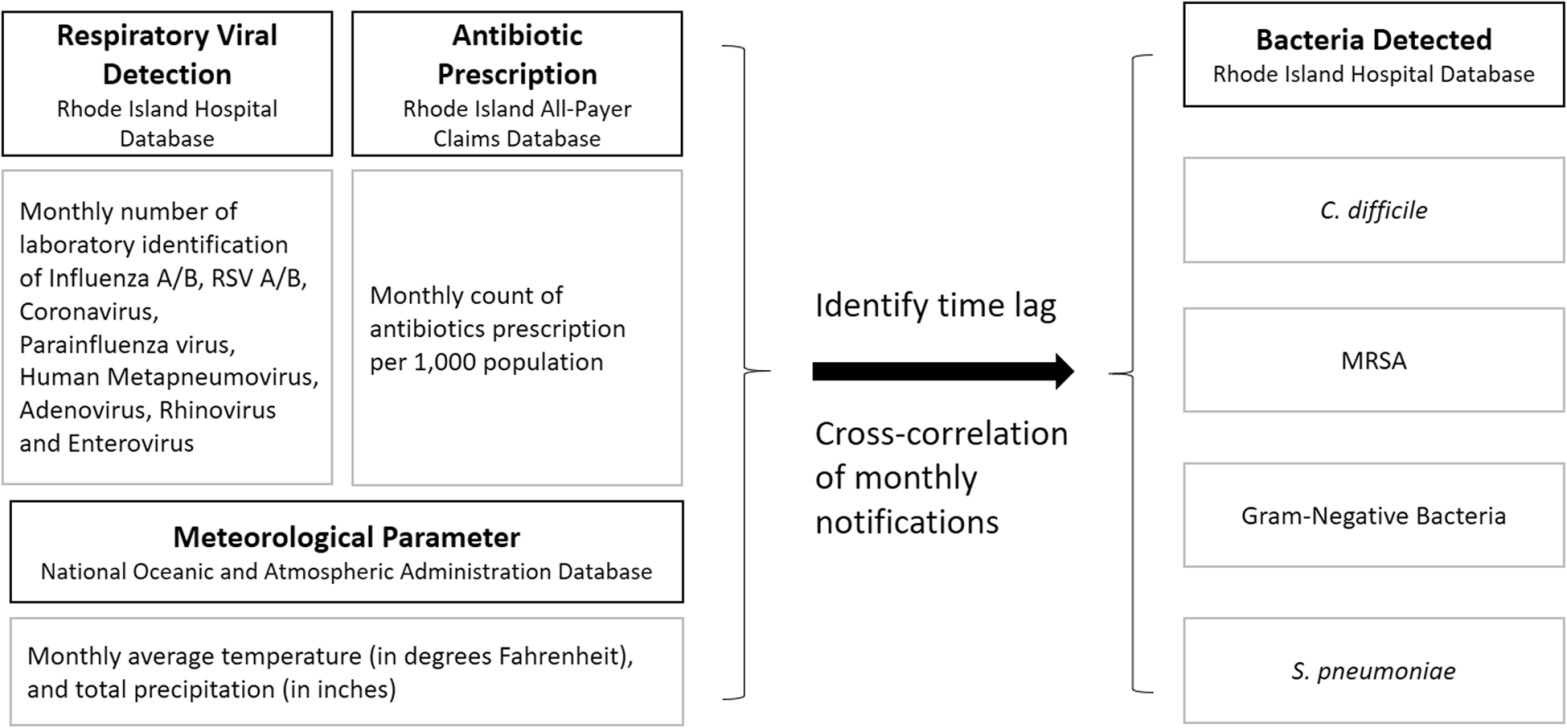
Schematic model for monthly distribution of respiratory virus detection, antibiotic prescriptions filled, and meteorological parameters (temperature and precipitation) correlating with detection of *Clostridioides difficile* in stool specimens, as well as methicillin-resistant *Staphylococcus aureus*, gram-negative bacteria, and *Streptococcus pneumoniae* in clinical isolates

The null hypothesis was no seasonal cross-correlation between respiratory viral activity, antibiotic prescriptions filled, weather variables and detection of different bacterial pathogens. We constructed a longitudinal incidence rate using a monthly dataset which included respiratory viruses detected, antibiotic prescriptions filled, and meteorological parameters (total precipitation and average temperature), as well as detected bacterial pathogens (*C. difficile*, MRSA, GNB, and *S. pneumoniae*) (Fig. 1). A seasonal trend decomposition procedure, based on Locally Weighted Scatterplot Smoothing (STL), was conducted for each bacterial pathogen to assess for seasonality and trends associated with detection of respiratory viruses, antibiotic prescription, and meteorological parameters [13]. An additive decomposition model was used. To assess for a correlation between a time series and a given a number of lags, we measured the cross-correlation of X_*t*_ and Y_*t + k*_ for each month. Separate cross-correlation functions were applied to determine the specific bacterial pathogens and their highest correlation with detection of respiratory viruses, antibiotic prescriptions filled, or meteorological parameters on defined time lags. We calculated odds ratios (OR) with 95% confidence intervals, estimating risk of elevated incidence compared to annual average incidence. Due to the small numbers and multiple testing, the *p*-values should only be seen as descriptive measures. Analyses were performed using R (ver. 3.4.3; R Development Core Team, Vienna, Austria). Packages used were: forecast, TSA (time series analysis) [14], and ASTA (applied statistical time series analysis) [15].

## Results

**Fig. 2:**
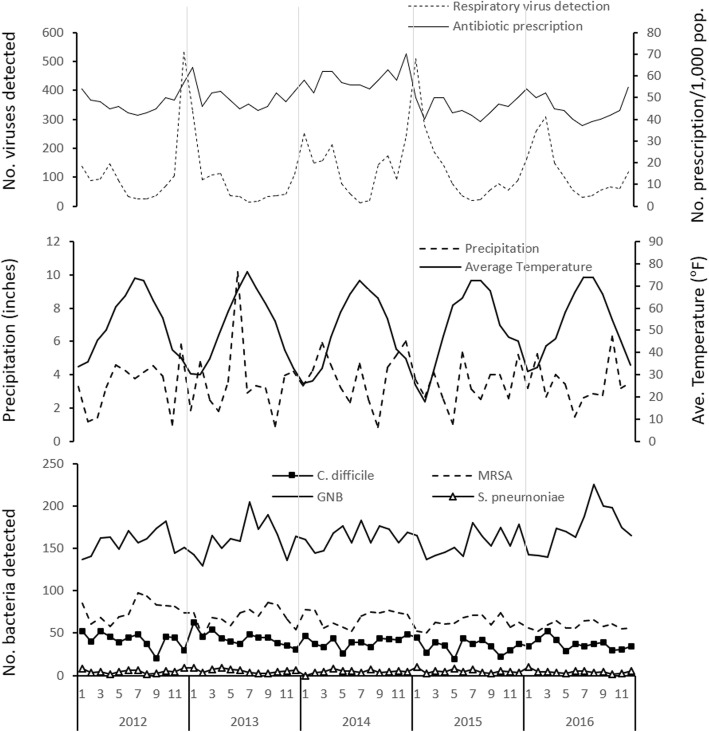
Time-trend decomposition of respiratory virus detection, antibiotic prescriptions filled, and meteorological parameters (temperature and precipitation)*,* correlated with detection of *Clostridioides difficile* in stool specimens, and detection of methicillin-resistant *Staphylococcus aureus*, gram-negative bacteria, and *Streptococcus pneumoniae* in clinical isolates per month

From January 2012 through December 2016, 6,857 respiratory viruses were detected and 3,065,789 antibiotic prescriptions were filled with distinct seasonality from December through February (Fig. 2-A). Peak precipitation and temperature were noted from June through August annually (Fig. 2-B). There were 16,419 bacterial pathogens identified: 2,354 *C. difficile*, 4,026 MRSA, 9,767 GNB, and 272 *S. pneumoniae* (Fig. 2-C). The level of detection of bacterial pathogens varied in month of onset, peak prevalence and duration of detection.

The peak in antibiotic prescriptions filled occurred 3 months before the peak in detection of *C. difficile* (OR = 1.24, 95% C.I., 1.07–1.43; *P* = 0.006; Table 1). Peak detection of MRSA occurred 7 months after the peak in antibiotic prescriptions filled (OR = 1.69, 95% C.I., 1.21–2.35; *P* = 0.003) and 10 months after the peak in respiratory virus detection (OR = 1.04, 95% C.I., 1.01–1.06; *P* = 0.003). The peak GNB detection occurred 2 months after the peak mean monthly temperature (OR = 1.69, 95% C.I., 1.20–2.39; *P* = 0.004). The peak in detection of *S. pneumoniae* occurred at the peak in detection of respiratory viruses (OR = 1.01, 95% C.I., 1.00–1.01; *P* = 0.015).

**Table 1:** Correlations between detection of respiratory viruses, antibiotic prescriptions, and meteorological parameters with detection of bacteria

Variables^a^	Lag (months)	Odds ratio	95% C.I.	Adjusted R^2^	*P*
*C. difficile*					
Antibiotic prescriptions	3	1.24	(1.07–1.43)	0.420	0.006
MRSA					
Respiratory virus	10	1.04	(1.01–1.06)	0.433	0.003
Antibiotic prescriptions	7	1.69	(1.21–2.35)	0.210	0.003
Gram-negative bacteria					
Temperature	2	1.69	(1.20–2.39)	0.131	0.004
*S. pneumoniae*					
Respiratory virus	0	1.01	(1.00–1.01)	0.270	0.015

^a^Cross correlations between detection of respiratory viruses, antibiotic prescriptions filled, and meteorological parameters (temperature and precipitation)-specific time series regression models with detection of *Clostridioides difficile* in stool specimens, as well as methicillin-resistant *Staphylococcus aureus* (MRSA), gram-negative bacteria, and *Streptococcus pneumoniae* detection in clinical isolates per month

## Discussion

Peak detection of *C. difficile* occurred a few months after the peak in antibiotic prescriptions during winter months, as previously described [7, 8]. Previous reports have also correlated increased *C. difficile* infections 1–2 months after peak macrolide and fluoroquinolone use [16].

We previously reported peaks of MRSA infections during the summer and autumn seasons [5]. This seasonality may, in part, be associated with increased antibiotic prescriptions some months earlier, as previously reported [17]. The 7-month lag that we detected is longer than found in another study which detected a temporal relationship between fluoroquinolone prescriptions and ciprofloxacin-resistant MRSA after a 1-month lag period [18]. A recent study revealed that antibiotics lacking MRSA activity, including fluoroquinolones, promote MRSA overgrowth in the nares and this may be related to finding increased MRSA detection in clinical isolates after exposure to such antibiotics [19]. Not surprisingly, a national antibiotic stewardship program has been associated with decreased MRSA prevalence [20]. We also found the circulation of respiratory viruses is likely associated with bacterial co-infection or superinfection due to *S. pneumoniae* (0-month lag with OR = 1.01) as previously reported [3].

Our findings are similar to a study of 132 US hospitals that found higher outdoor temperatures associated with an increased frequency of bloodstream infections caused by GNB [21]. A review of the published literature also revealed that increased temperature correlated with bloodstream infections due to GNB [4]. A study from Brazil found that average temperature on the day of diagnosis was associated with *Klebsiella* spp. (OR 1.19; 95% CI 1.07–1.33) and *A. baumannii* (OR 1.20; 95% CI 1.07–1.34) [22]. However, this finding may not be replicated in different geographic locations with varying weather conditions [2]. The reasons for this association are unclear but likely, in part, reflects some advantage in environmental growth conditions with warmer temperatures among various GNB. It is possible that seasonal changes in the human microbiome plays a role [23].

There are limitations of this study. We collected de-identified data using infection control software rather than detailed medical record data. Misclassification bias may be present because we could not exclude clinically-insignificant microbial colonizers or bystanders which may have overestimated the number of true infections. We may have had selection bias since the microbiologic data was collected within our hospital system and outpatient facilities served by our microbiology laboratory and this data may not be reflective of all Rhode Islanders. Respiratory viral infections in children could have a different seasonal pattern compared to adults. There may be detection bias since healthcare providers may not have thought of ordering tests for respiratory virus detection outside of the winter season. In addition, there may have been a change in the denominator population captured over the time course that could potentially influence these findings. Since this is an ecological study, the temporal association can be easily confounded with other factors that are seasonal such as duration of antibiotics and the level of humidity. Viruses and bacteria in this study were detected in patients in one of our three acute care hospitals which comprises 55% of non-Veteran’s Administration medical center acute care beds in Rhode Island, and at various outpatient settings in Rhode Island. Antibiotic prescription data was derived from a database that included people throughout Rhode Island, some of whom were not cared for in our healthcare system. Thus, we cannot associate antibiotic prescriptions filled and microbiologic test results on an individual level. Despite these potential limitations, we are not aware of other studies that assessed the relationships among bacterial pathogens, respiratory viruses, antibiotic exposure, and meteorological parameters in the same geographic region.

## Conclusion

In conclusion, we attempted to study the complex ecology of bacterial and viral infections, and assess the potential impact of antibiotic exposure and climactic fluctuations. The temporal association between antibiotic prescriptions filled with detection of *C. difficile* highlights the potential impact of robust antibiotic stewardship on reducing *C. difficile* risk. Prevention of respiratory viral infections may reduce risk of *S. pneumoniae* infections as previously described. A better understanding of the seasonality of GNB and MRSA may help to focus future infection prevention efforts. As such, continued research is needed to better understand why these infections follow seasonal patterns.

## Additional file

Additional file 1: **Figure S1.** Trend of positive respiratory viral panel (RVP) testing over time. (PNG 30 kb)

## Data Availability

Not applicable.

## Abbreviations

ACPD: All Payer Claim Database
ASTA: Applied statistical time series analysis
GNB: Gram-negative bacteria
MRSA: Methicillin-resistant *Staphylococcus aureus*
RSV: Respiratory syncytial virus
STL: Locally Weighted Scatterplot Smoothing
TSA: Time series analysis
