# Supplementary figures and images for: Seasonality of respiratory viruses and bacterial pathogens
Source: Antimicrob Resist Infect Control. 2019 Jul 22;8:125. doi: 10.1186/s13756-019-0574-7 (PMC6647268; doi:10.1186/s13756-019-0574-7)

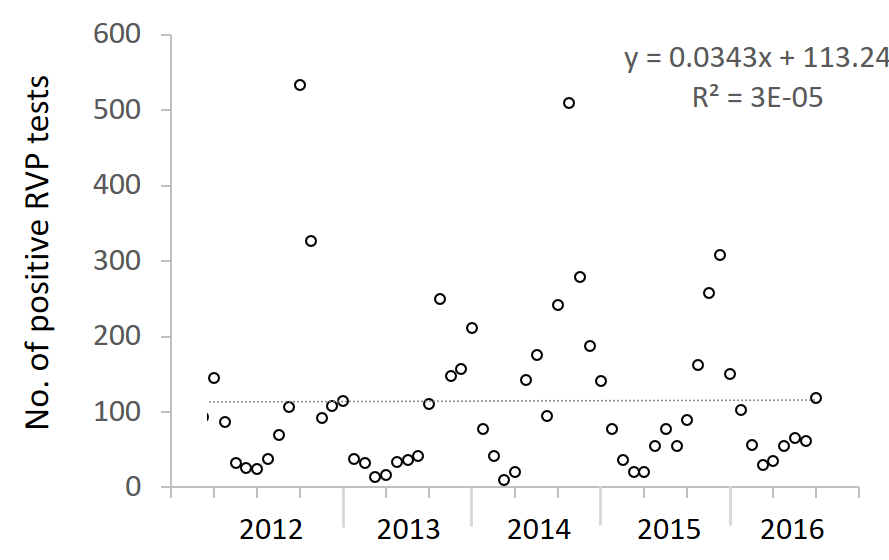

Supplement: Supplementary file 1 — Figure S1. Trend of positive respiratory viral panel (RVP) testing over time. (PNG 30 kb) [file 13756_2019_574_MOESM1_ESM.png]
